# Spatiotemporal Dysfunction of the Vascular Permeability Barrier in Transgenic Mice with Sickle Cell Disease

**DOI:** 10.1155/2012/582018

**Published:** 2012-06-12

**Authors:** Samit Ghosh, Fang Tan, Solomon F. Ofori-Acquah

**Affiliations:** ^1^Aflac Cancer and Blood Disorders Center, Division of Hematology/Oncology/BMT, Department of Pediatrics, Emory University School of Medicine, Atlanta, GA 30322, USA; ^2^Department of Pediatrics, Children's Healthcare of Atlanta, Atlanta, GA 30322, USA

## Abstract

Sickle cell disease (SCD) is characterized by chronic intravascular hemolysis that generates excess cell-free hemoglobin in the blood circulation. Hemoglobin causes multiple endothelial dysfunctions including increased vascular permeability, impaired reactivity to vasoactive agonists, and increased adhesion of leukocytes to the endothelium. While the adhesive and vasomotor defects of SCD associated with cell-free hemoglobin are well defined, the vascular permeability phenotype remains poorly appreciated. We addressed this issue in two widely used and clinically relevant mouse models of SCD. We discovered that the endothelial barrier is normal in most organs in the young but deteriorates with aging particularly in the lung. Indeed, middle-aged sickle mice developed pulmonary edema revealing for the first time similarities in the chronic permeability phenotypes of the lung in mice and humans with SCD. Intravenous administration of lysed red blood cells into the circulation of sickle mice increased vascular permeability significantly in the lung without impacting permeability in other organs. Thus, increased vascular permeability is an endothelial dysfunction of SCD with the barrier in the lung likely the most vulnerable to acute inflammation.

## 1. Introduction

Sickle cell disease (SCD) is characterized by the production of red blood cells with increased propensity for lysis and adhesion [1]. Its clinical manifestations fall broadly into two subphenotypes defined by hyperhemolysis and vasoocclusion [2]. At least 30% of the hemolysis in SCD is intravascular [3], which means that the endothelial wall in this disease is persistently exposed to cell-free hemoglobin. The endothelium is a semipermeable barrier that regulates the response of the vascular wall to inflammatory agonists. This response involves activation of adhesion molecule expression, increased permeability of the endothelium, and extravasations of fluid from the blood into interstitial tissue compartments [4]. Increased vascular permeability results from opening of gaps at sites of endothelial cell-cell contacts. There are multiple indicators of systemic inflammation in SCD [5]. In addition, markers of vascular inflammation have also been documented [6–8]. There is increased expression of adhesion molecules in the pulmonary endothelium of the Berkeley sickle mice [9], although the histology of these same mice shows less severe inflammatory and ischemic changes and no evidence of pneumonia [10]. Nonetheless, they spontaneously develop pulmonary hypertension [11], which is a major problem in SCD [12]. Pulmonary edema and the acute chest syndrome implicate increased vascular permeability in both chronic and acute complications of SCD [13, 14]. Despite this significance, there is currently no knowledge of the vascular permeability phenotypes of major organs that are impacted by SCD.

## 2. Materials and Methods

### 2.1. Transgenic Sickle Mice

Experiments were performed using protocols approved by the Institutional Animal Care and Use Committee (IACUC) of Emory University. The Berkeley [15] and Townes [16] transgenic SCD mouse models used have previously been described. 

### 2.2. Vascular Leakage and Lung Edema

Vascular leakage was studied by intravenous injection of cell-impermeable Evan's Blue dye as widely described by several investigators. Mice were injected with 100 *μ*L of 1% cell-impermeable Evans Blue dye (Sigma-Aldrich, St. Louis, MO) in PBS intravenously through tail vein. After 40 min, mice were anesthetized by i.p. injection of avertin (300 mg/kg body weight). To remove the dye from circulation, mice were perfused by injecting 40 mL of PBS containing 2 mM EDTA through left ventricle of the heart allowing the blood to flow out by puncturing renal artery. Organs were harvested and incubated in formamide for 3 days to extract the dye and OD determined at 620 nm. For edema analysis mice were euthanized and the right lobe removed and weighed immediately using an isometric transducer (Harvard Apparatus, Holliston, MA). Lungs were dried in an oven at 80°C containing desiccant crystals for 24 h, dry weight determined, and ratios calculated. 

### 2.3. Statistical Analyses

GraphPad software version 5.0 was used. Differences in vascular leakage and weights were analyzed using *t*-test and correlation studies performed using Pearson's test.

## 3. Results and Discussion

**Figure 1:**
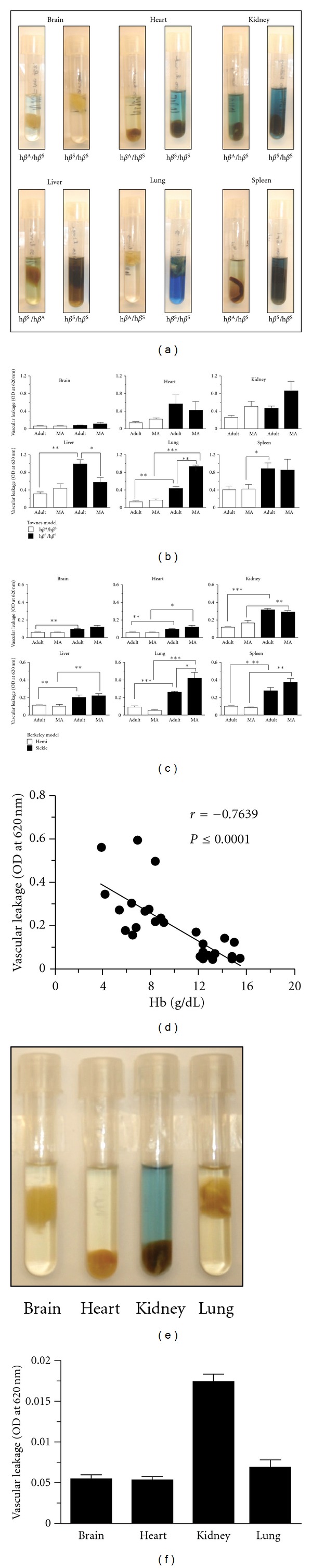
Vascular barrier dysfunction in sickle mice. (a) Representative images of organs isolated from sickle mice after injection with Evans blue dye and incubation in formamide for three days. (b, c) Vascular leakage in the indicated organs in adult (3–6 months) and middle-aged (10–13 months) mice of the Townes (heterozygotes-HbAS (*hβ* ^A^/*hβ* ^S^) and homozygote sickle-HbSS (*hβ* ^S^/*hβ* ^S^)) and Berkeley (hemizygotes and sickle) models. The number of mice studied was as follows: Townes: *n* = 3 for each genotype and age group; Berkeley: Sickle adult, *n* = 9; sickle middle-aged *n* = 6; hemizygote adult, *n* = 10, hemizygote middle-aged *n* = 5. (d) Vascular leakage correlates with hemoglobin. Data shown is the vascular leakage in the lung for a total of 30 mice (15 sickle and 15 hemizygotes) of the Berkeley model. (e) Typical images for the indicated organs isolated from young (4–6 weeks old) Berkeley mice injected with Evans blue dye and incubated in formamide for 3 days. (f) Histogram showing the quantification of Evans blue extravasation of major organs in young sickle mice (*n* = 4). *P < 0.05, **P < 0.01, and ***P < 0.001.

In SCD, the adhesive and vasomotor defects of the vasculature are well defined [17–20], while the vascular permeability remains poorly appreciated. To address this knowledge gap, adult (3–6 months) and middle-aged (10–13 months) mice were injected with 1% Evans blue via the tail vein and the amount of dye that leaked from the circulation into the parenchyma of individual organs examined.  Figure 1(a) shows virtually no leakage in the brain contrary to the clear evidence of endothelial barrier breakdown in the other organs. Quantification of vascular leakage revealed that the endothelial barrier is generally more permeable in the sickle mice than in control littermates (Figures 1(b) and 1(c)), despite some differences in the two transgenic models. Indeed, there was a significant correlation between steady-state hemoglobin concentration and lung permeability (Figure 1(d)) (*r* = −0.7639, *P* < 0.0001), indicating that endothelial barrier dysfunction is related to an aspect of SCD. Unlike most organs, vascular permeability in the lung in middle-aged sickle mice was significantly higher than in adult mice, highlighting a role for age in this disease process. This was investigated by extending our study to include younger mice aged 5-6 weeks. Remarkably, permeability in the heart and lung at this early stage of the disease was identical to that of the brain, which is widely known to have a highly restrictive barrier (Figures 1(e) and 1(f)). Thus, the endothelial barrier in SCD is normal in most organs in the young, becomes abnormal during adulthood, and deteriorates further with aging particularly in the lung. 

**Figure 2:**
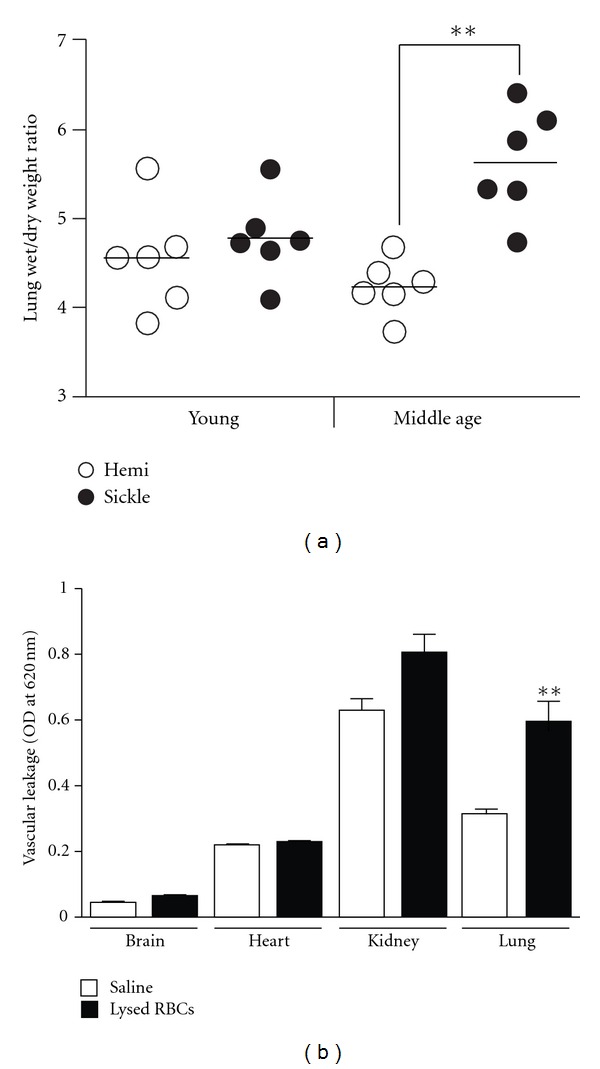
Chronic and acute changes in vascular permeability in sickle mouse lungs. (a) Lung edema in middle-aged Berkeley sickle mice as determined by wet/dry weight ratios (*n* = 6). Control groups include young sickle mice (*n* = 6) and young (*n* = 6) and middle-aged (*n* = 6) hemizygotes. (b) Vascular leakage in the indicated organs in Berkeley sickle mice intravenously injected with lysed red blood cells. Note that permeability is significantly increased by lysed red blood cells in the lung but not in other organs. **P < 0.01.

Pulmonary edema is a common postmortem finding in SCD and yet it has not been appreciated as a chronic lung complication of SCD, probably because of the confounding effect of death [21, 22]. While histology does not reveal evidence of edema in the Berkeley sickle mice [10], we have used a more sensitive approach to clearly demonstrate that vascular permeability in the lungs of both the Berkeley and Townes sickle mice is increased. Our result is in agreement with a recent study that investigated permeability exclusively in the lung of 12–16-weeks-old NY1DD sickle mouse using the same approach [23]. In agreement with these permeability findings, we discovered that the average wet lung weight of middle-aged sickle mice is significantly heavier than that of age-matched hemizygotes (0.64 mg ± 0.04 versus 0.54 mg ± 0.02; *P* = 0.03), and, accordingly, the wet/dry weight ratio, widely used to confirm edema in lungs, was significantly higher (*P* = 0.002) (Figure 2(a)). Taken together, these results show for the first time that middle-aged sickle mice develop pulmonary edema. We cannot exclude the possibility that chronic heart failure contributed to the lung edema reported here; however, the Berkeley mice used in the lung weight measurements were at least three months younger than those reported to have heart failure [11]. Importantly, we show that lung edema correlates with higher vascular permeability in sickle mouse lungs. This concordance advances both conceptual and practical research objectives. It suggests that the permeability phenotypes of the lung in mice and humans with SCD are similar, and it validates, for the first time, the use of the Evans blue extravasation approach to study vascular permeability in transgenic sickle mice. 

The permeability phenotypes identified here likely reflect the intrinsic properties of individual organs, as well as of endothelial cell types. For instance, it is well established that heterogeneity of endothelial cell junction contributes to unique permeability attributes [24], and this may account for some of the dramatic differences in permeability phenotypes reported here (e.g., brain and lung). However, endothelial cells in individual organs may also respond differently to barrier disrupting factors found in SCD. Among these factors, cell-free hemoglobin is unique because it is released in abundance during acute intravascular hemolysis, which is a daily event in SCD. We assessed the response of major vascular beds in the sickle mice to acute hemolysis. Leuko-depleted packed red blood cells were lysed by repeated freeze-thaw cycles and intravenously administered to sickle mice via tail vein. Compared to saline, the lysed red blood cells increased vascular permeability by 2-fold in the lungs of adult sickle mice but had a modest or negligible impact on the kidney, brain, and heart (Figure 2(b)). That lysed red blood cells caused morbidity and significantly altered barrier function in the Berkeley sickle mice indicates this and other severe models of SCD can be used to unlock mechanisms of acute hemolysis in SCD. In particular the vulnerability of the lung endothelial barrier to lysed red blood cells highlights acute intravascular hemolysis as a potential trigger of the acute chest syndrome since a decreasing concentration of hemoglobin is invariably associated with this condition [14]. 

In conclusion, SCD appears to be characterized by weakening of the endothelial barrier, which predisposes some organs, such as the lung to acute loss of barrier function, reminiscent of the acute chest syndrome. Ongoing studies are focused on unraveling the relationship between acute hemolysis and the endothelial barrier in SCD.
